# Resilience and Protection of Health Care and Research Laboratory Workers During the SARS-CoV-2 Pandemic: Analysis and Case Study From an Austrian High Security Laboratory

**DOI:** 10.3389/fpsyg.2022.901244

**Published:** 2022-07-22

**Authors:** Martina Loibner, Paul Barach, Stella Wolfgruber, Christine Langner, Verena Stangl, Julia Rieger, Esther Föderl-Höbenreich, Melina Hardt, Eva Kicker, Silvia Groiss, Martin Zacharias, Philipp Wurm, Gregor Gorkiewicz, Peter Regitnig, Kurt Zatloukal

**Affiliations:** ^1^Diagnostic and Research Institute of Pathology, Medical University of Graz, Graz, Austria; ^2^College of Population Health, Thomas Jefferson University, Philadelphia, PA, United States; ^3^School of Medicine, The University of Queensland, Brisbane, QLD, Australia

**Keywords:** SARS-CoV-2, COVID-19 pandemic, research laboratory workers, biosafety level-3 (BSL-3), personal protective equipment (PPE), occupational challenges

## Abstract

The SARS-CoV-2 pandemic has highlighted the interdependency of healthcare systems and research organizations on manufacturers and suppliers of personnel protective equipment (PPE) and the need for well-trained personnel who can react quickly to changing working conditions. Reports on challenges faced by research laboratory workers (RLWs) are rare in contrast to the lived experience of hospital health care workers. We report on experiences gained by RLWs (e.g., molecular scientists, pathologists, autopsy assistants) who significantly contributed to combating the pandemic under particularly challenging conditions due to increased workload, sickness and interrupted PPE supply chains. RLWs perform a broad spectrum of work with SARS-CoV-2 such as autopsies, establishment of virus cultures and infection models, development and verification of diagnostics, performance of virus inactivation assays to investigate various antiviral agents including vaccines and evaluation of decontamination technologies in high containment biological laboratories (HCBL). Performance of autopsies and laboratory work increased substantially during the pandemic and thus led to highly demanding working conditions with working shifts of more than eight hours working in PPE that stressed individual limits and also the ergonomic and safety limits of PPE. We provide detailed insights into the challenges of the stressful daily laboratory routine since the pandemic began, lessons learned, and suggest solutions for better safety based on a case study of a newly established HCBL (i.e., BSL-3 laboratory) designed for autopsies and research laboratory work. Reduced personal risk, increased resilience, and stress resistance can be achieved by improved PPE components, better training, redundant safety measures, inculcating a culture of safety, and excellent teamwork

## Introduction

The COVID-19 pandemic has heavily burdened the world’s population, health care systems’ and research organizations’ staff all over the world, and various occupational groups in varying ways. Initially little was known about how to deal with a pandemic of this magnitude. SARS-CoV-2 has been classified as a risk group-3 pathogen by the world health organization (WHO) and centers for disease control (CDC) due to its high infectivity, mortality rate, and the fact that no fully effective treatment is available, and therefore requires specific biosafety measures (Kaufer et al., 2020; Schröder, 2020; CDC, 2021).

Health care workers (HCWs) in hospitals are a particularly mentally and physically challenged occupational group at high risk for infection with SARS-CoV-2. “*The WHO estimates that between 80,000 and 180,000 health and care workers could have died from COVID-19 in the period between January 2020 and May 2021, converging to a medium scenario of 115,500 deaths*” (WHO, 2021).

## Awareness of the Risks and Hazards to Laboratory Workers

In contrast to several publications on the marked challenges that COVID-19 caused for HCW (Van Zundert et al., 2020), very little attention has been paid to laboratory workers in High-Containment Biological Laboratories (HCBL). We describe the role of laboratory workers in the pandemic and report on our experiences obtained in a biosafety-level-3 (BSL-3) laboratory in Austria that is actively involved in a broad spectrum of diagnostic and research work related to COVID-19 management.

Diagnostic laboratory workers performed massive amounts of antigen and PCR tests under BSL-2 conditions while wearing cumbersome personal protective equipment (PPE) including N-95 masks or higher-level respirators, eye protection, gloves, and gowns (CDC, 2021). Research work required cultivation and propagation of the virus which leads to higher virus concentrations than in patient samples and must therefore be carried out under BSL-3 conditions. The global labor capacity of BSL-3 laboratories is limited in terms of space, equipment and trained research laboratory workers (RLWs). As a consequence RLWs were required to work multiple and exceptionally long shifts (up to 9 h) to meet the enormous demand for generating basic knowledge of SARS-CoV-2 and its interaction with infected patients as well as to develop diagnostic tests, preventive (e.g., vaccines) and therapeutic (e.g., drugs) solutions. For example, RLW in the BSL-3 laboratory of the Medical University Graz, Austria performed experimental work essential for developing antigen- or PCR-based diagnostic tests, vaccines by testing the virus neutralizing activity generated by vaccination, and virus inhibiting substances, such as natural substances, small chemical molecules or biologics in multiple experimental setups as well as testing new decontamination technologies contributing to managing the pandemic (Table 1). Since there was a global shortage in BSL-3 capacities to perform this type of research work the COVID-19 pandemic resulted in an unprecedented workload on RLWs in BSL-3 labs. Nevertheless, there are only few reports on the broad spectrum of occupational challenges and risks that were faced by this group of RLWs.

**TABLE 1:** Major laboratory activities and experimental tasks performed by medical, diagnostic, and scientific research laboratory workers in context of SARS-CoV-2.

*BSL-2* with additional personal protective equipment (PPE): handling patient samples, e.g., nasopharyngeal swabs, saliva, gargle solutions
Performance of diagnostic tests (PCR, antigen)
*BSL-3* with additional PPE: propagative virus samples, virus cultivation, *in vitro* assays
Development and validation of diagnostic tests (PCR, antigen)
Evaluation of virus properties
Stability on various materials, porous (masks, fabrics) and non-porous (metal, synthetic material, coatings, nanomaterial)
Development of novel decontamination approaches
Efficacy of disinfectants
Reduction of environmental risks
Decontamination of PPE, laboratory devices, utility items
Development of re-use processes for PPE
Basic scientific questions investigating immunology and virology of SARS-CoV-2
Investigation of virus behavior in cell lines and animal models
Drug and Vaccine development
Assays for virus inhibition with various substances, drugs, repurposing of already approved medicines, convalescent plasma, (artificial) antibodies, natural substances, vaccine candidates in cell culture and animal models
Experimental approval procedures for substances and vaccines to fulfill regulatory requirements for subsequent clinical trials
Preparation of various *in vitro* results for diagnostic, therapeutic and vaccine clinical trials for patients
Autopsies
Investigation of distribution and impact of the virus on the whole body and various tissue types
Sample preparation and comprehensive tissue analyses to investigate virus-induced cell and tissue damages
Evaluation of comorbidities
Basis for rapid and fact-based risk management for decision about preventive measures
Biobanking
Acquisition, storage and management of patient’s specimens (BSL-2 and BSL-3) and samples cultivated therefrom (BSL-3)

## Morbidity, Vulnerability, and Resilience of Health Care Workers and Research Laboratory Workers During COVID-19

HCWs are considered to be at the highest risk for infection due to direct exposure, limited availability of PPE, breaches and non-adherence to infection prevention and control (IPC) protocols (WHO, 2020a). While the benefits of the PPE are well known, the taxing working conditions cause great physical, operational and psychological stress (Yuan et al., 2020b; Bonell et al., 2021; Davey et al., 2021; Galanis et al., 2021; Messeri et al., 2021).

The impact on different population and professional groups’ vulnerabilities and resilience factors became gradually visible early in the pandemic. This has been evaluated in several surveys and questionnaires, mostly of HCWs (Abtahi et al., 2021; Ambrose et al., 2021). The PubMed search of terms “SARS-CoV-2 AND workload AND health care workers” yielded 202 publications as of February 8th 2022. The terms “SARS-CoV-2 AND workload AND laboratory workers” yielded 19 publications, but only 3 publications refer to RLWs. Massive workload, heat stress, skin irritation, burnout, moral injury, depression, and self-injury, daily exhaustion mostly due to the use of PPE, fear of infection and contagion of one’s family members, extended working shifts due to sick colleagues or being in quarantine are stressors reported to be the greatest burdens on HCWs (Bonell et al., 2021; Catalán et al., 2021; De Kock et al., 2021; Galanis et al., 2021; Messeri et al., 2021). A recent cross-sectional study of HCW indicated a 15% increase in the demand for mental healthcare professional support in the first year of the pandemic (Saunders et al., 2022). Our review of the literature showed either no distinction in principle between HCWs and different RLWs, and the majority of stress studies only applied to frontline HCWs with direct contact with infected patients. One exception is an Italian field study with 635 HCWs (195 nurses, 147 physicians, 158 technicians, 135 administrative personnel), and outlined the psychological needs and excessive workloads on HCWs. The authors specifically discovered that the professional category of technicians had significantly demonstrated a double risk for sleep problems and chronic fatigue as well as a three-fold risk for reduced capacity to recover and return to work. This was reported to be the most frequent cause of lasting psychological impairment, affecting about a half of the studied population, followed by sleep problems found in 44.7% (Di Prinzio et al., 2021).

De Kock et al. (2021), Grossman (2021), and Giorgi et al. (2020) state that various strategies to overcome the pandemic’s implications which are adapted to the different and individual’s requirements still need to be examined, developed and implemented. However, the psychological impact of SARS-CoV-2 in a specific, vulnerable and mostly “hidden group” of (diagnostics) RLWs, who are required to handle infected patients’ blood samples at a high-risk exposure to SARS-CoV-2, was described by Teo et al. (2021). In addition they highlighted the increased workload and psychological needs of RLW (Teo et al., 2021).

## Significance of High-Containment Biological Laboratories During the Sars-CoV-2 Pandemic

High-Containment Biological Laboratories (HCBLs; e.g., BSL-3 and BSL-4 laboratories) play a critical role in the rapid advancement of research to characterize human and animal high-risk pathogens, assist in disease surveillance, and conduct the initial pre-clinical research that sustains the pipeline for development of diagnostics, therapeutics and vaccines. Scenarios involving work with high titer virus cultures, potential exposure to aerosols, divergent high transmission variants, and zoonosis from laboratory animals require at a minimum BSL-3 conditions. HCBLs have evolved in terms of infrastructure, space, physical controls, policies, human resources, and workforce training (Casanova et al., 2017). Establishing HCBLs is costly and needs continuous investment in resources and personnel to sustain safe labor, equipment, infrastructure, certifications, and operational needs. A broad spectrum of redundant biosafety and biosecurity precautions is implemented in physical containment facilities that operate under negative air pressure and have air locks as well as waste deactivation systems to minimize the risk to RLW of laboratory-acquired infections and protect the environment. This has to be complemented by periodic training of personnel and dedicated risk management, practices and protocols involving risk-based assessments to ensure biosafety and biosecurity in HCBLs when performing tasks with agents and material such as new and emerging viruses (International Organization for Standardization IOS, 2019; CDC, 2020).

## Analysis and Case Study -BSL-3 Laboratory: Medical University Graz, Austria

The BSL-3 laboratory was built at the new campus of the Medical University of Graz and became operational in December 2019. It experienced all the developmental challenges that a new HCBL and its employees face at the start of operations. The BSL-3 laboratory is associated to the Diagnostic and Research Institute of Pathology and the teams working in the laboratory consist of molecular scientists (12), pathologists (5) and autopsy assistants (3). They perform a myriad of work with SARS-CoV-2 such as autopsies (Zacharias et al., 2021), frozen section diagnostics, and sample preparations for molecular pathology covering the routine tasks of diagnostic pathology. Specimen collection for further investigations and biobanking (tissues, swabs, body fluids), isolation and cultivation of virus, and research work as described in Table 1 but excluding animal experiments. Collaboration with groups at the Diagnostic and Research Institutes of Pathology and of Hygiene, Microbiology and Environmental Medicine, complements the medical and scientific expertise and provides genetic sequencing capacities.

**FIGURE 1:**
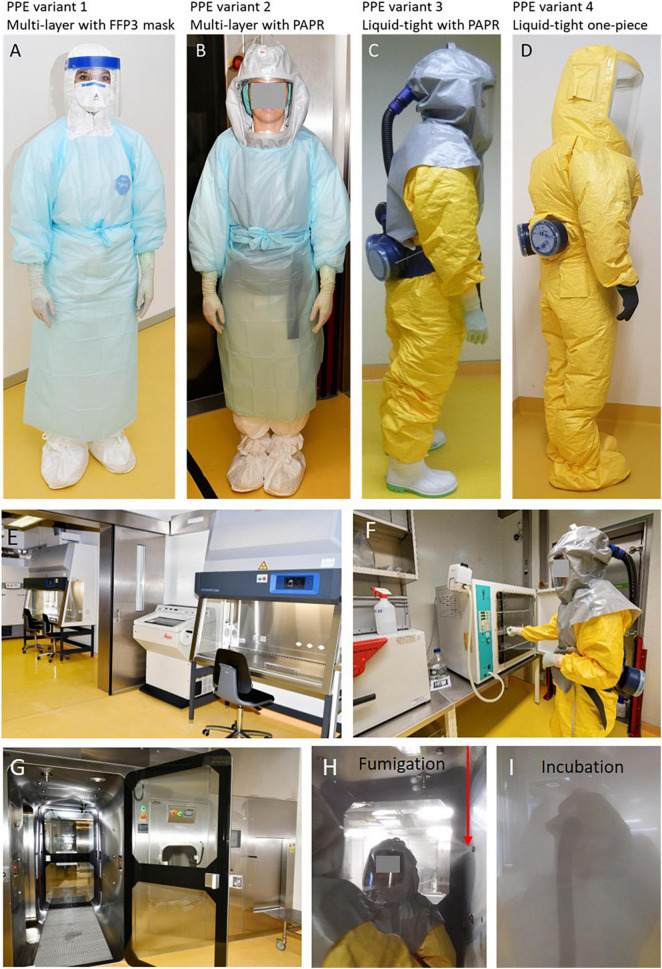
(A) Personal protective equipment (PPE) variant 1 consists of a single-use coverall (Cat. III, type 4-B/5-B/6-B), apron with long sleeves, a double layer of shoe covers worn over crocs, a double layer of differently colored gloves, e.g., green nitrile gloves as the inner layer and white gloves as the outer layer, FFP3 mask, goggles and face shield. (B) PPE variant 2 corresponds with variant 1 but FFP3 mask, goggles and face shield are replaced by a powered air-purifying respirator (PAPR, e.g., Jupiter™ Air Filter Unit with two A2BEKP filters and 8 h rechargeable batteries) connected to a hood assembly providing head, face and shoulder coverage. (C) PPE variant 3 consists of a Tychem 2000C protective suit (Cat. III, Type 3/4/5/6) which protects against biological substances, and is impermeable to liquid and particles. Outer layers of gloves and chemical protective boots are sealed with adhesive tape. The hood is ventilated by the same PAPR used in variant 2. (D) PPE variant 4 consists of a one-piece Tychem ProChem protective suit (CPM F1 H L2, Cat. III, Type 3/5) with integrated boot socks and gloves, resistant against biological hazards, particles, and liquids. (E) Two biosafety cabinets category II connected to the exhaust air allow parallel work with different agents. (F) Incubators for cell culture and bacteria. (G) The chemical shower is an additional safety device for decontamination of corpses in a body bag after autopsies and lab workers by means of different processes and disinfectants. (H) Disinfectants are fumigated through eight nozzles (red arrow) into the shower cabinet and (I) incubated for a defined time period. RLWs must move to ensure safe distribution of the disinfectant.

The laboratory design was based on experiences gained by participating in the preparatory phases of the European Research Infrastructure on Highly Pathogenic Agents (ERINHA^1^), which includes most European BSL-4 and BSL-3 laboratories. We have implemented several biosafety features of BSL-4 laboratories e.g., a chemical shower to decontaminate personnel and corpses after autopsies, and biosecurity measures (Loibner et al., 2021a; Figures 1E–I).

Before the laboratory became operational we conducted a study (ClinicalTrials.gov, NCT03004690) with nineteen volunteers wearing head- or full body-ventilated PPE suits with powered-air-purifying-respirators (PAPR) to select the appropriate PPE components and assess the usability and limiting factors. The volunteers handled four tasks during six working hours at 22°C and four working hours at 28°C on another day in a mock-up HSBL. The most constraining factors caused by PPE were reduced hand dexterity due to multiple glove layers, impaired visibility due to flexible face shields and back pain due to the heavy respirator in the fully ventilated suit. Heat stress and liquid loss were reported as restrictive at a working temperature of 28°C but were well tolerated at 22°C. However, these factors had no negative impact on the general work performance, reaction time and error rates (Loibner et al., 2019). The respective PPE components for different variants (Figures 1C,D) were readily available before the pandemic, and were stockpiled for further trainings and decontamination process validation. When the pandemic began, this preparatory work enabled a good response to the sudden high requirements of PPE components due to the rapid increase in workload and the emerging additional operational limitations in certain PPE variants (Loibner et al., 2021b).

Our lab has gained much experience and detailed knowledge since March 2020 on the advantages and limitations of PPE components by performing more than 50 COVID-19 autopsies. Lessons learned showed that PPE variant 1 with the FFP3 mask (Figure 1A) led to mask fogging and slipping of the eye glasses and goggles during prolonged PPE use, which led to one injury when working with a knife. The constricted feeling from long-term wearing of FFP3 masks in combination with the reduced oxygen supply and dehydration due to sweating led to anxiety and circulatory problems for one team member. Furthermore, this PPE variant protection was considerably compromised by increasing beard length (Prince et al., 2021). As a consequence, we decided to use PPE variant 2 (Figure 1B) with a hood and PAPR, which allows for normal breathing, reduces heat stress, and prevents unintentional face and neck touching. PPE variant 1 was subsequently used only for autopsies of patients with Creutzfeldt-Jacob disease because the PPE components cannot be decontaminated and were incinerated (which is not an option for PPE with PAPR).

In parallel to performing autopsies the laboratory was also intensively used for a broad spectrum of research work (Table 1), mostly using PPE variant 3. Lessons learned with this PPE variant demonstrated a variety of shortcomings, requiring improvement and development of innovative PPE components in the future. For example, the ribbons of the hood for the PAPR easily tore off which then required provisional and improvisational repair using adhesive masking tapes. PPE variant 4 (Figure 1D) does not allow a battery change of the PAPR, which might be required during long working shifts (>4–6 h), and the integrated shoes are more slippery than the PPE variant 3 with rubber boots (Figure 1C). We also experienced different types of malfunctions of the PAPR including blower motor failure and breaking of the filter fittings that required emergency exit from the laboratory.

Usually in the Graz BSL-3 laboratory, rest or bathroom breaks during a working shift are not performed. In order to keep the time in the laboratory as short as possible experiments are precisely planned, reaction times during experiments are optimally used, and different experimental procedures are nested to reduce working time. Nevertheless, during the critical phase of the pandemic, working shifts of more than 8 h were required showing not only the individual but also the ergonomic and safety limits of certain PPE variants.

Additionally, a general problem we found was that the liquid-tight coveralls (Tychem material) were not available in small sizes, especially for petite women with heights less than 165 cm. Due to the massive wrinkle formation, the wearing comfort and also the effects of the decontamination were impaired. Individual sizing would significantly increase the wearing comfort and support sustained clinical operations. Furthermore, proper sizing of PPE can reduce the risk of damaging the coverall, which happened twice due to sharp edges of the laboratory equipment.

Our laboratory faced major problems due to interrupted supply chains for key PPE components and chemical reagents. In this context the availability of the chemical shower was essential because, during the shortage of single-use coveralls and FFP3 masks, we switched to using the more expensive liquid-tight coveralls and the PAPR. The combination availability of these back up options for repeated (off-label) re-use of single-use coveralls within the certified maximal wearing time, was essential to stay operational. The chemical shower also minimized the risk of self-contamination during the doffing process, which is known to pose a major risk for contaminating and infecting personnel (Chughtai et al., 2018).

At the beginning of the pandemic, there were significantly fewer routine diagnostic activities due to the sudden stop of public life, so the BSL-3 area was more readily available and autopsies could be carried out without delays. The avoidance of time stress when donning and doffing, and during autopsy activities, represent the critical safety factors in avoiding PPE breach and contamination. Strict compliance with the security measures resulted in a good sense of security and the focus was on clarifying the consequences of the COVID-19 infection.

We learned that no matter which PPE variant was used, mutual supervision by other colleagues was necessary for safe donning and doffing. The buddy oversight enabled better following of the respective protocols and checklists, and mutual control of the equipment for completeness and intactness in mitigatating risks and errors. This helped to boost confidence in the PPE during the COVID pandemic, which has exposed a serious HCW trust gap in management around the world (Auerbach et al., 2021). Mirrors in the locker room and the adjacent airlock room facilitated the correct donning of multi-layer PPE variants, as recommended by Ruskin et al. (2021).

In addition to the human factors, other technical issues can create hazardous situations. We learned that the impact of combinations of failures can hardly be predicted. We performed emergency trainings with a focus on combined failures of different systems and incidents, such as fire, energy failure and need of rescue and decontamination of an injured person through the emergency exit. This led to the further specification and improvement of the physical design and flow of work, and the fine tuning of emergency protocols (Capolongo et al., 2020). Simple, low-cost environmental design interventions can provide support and improve HCW and RLW safety in biocontainment units (DuBose et al., 2018; WHO, 2021). Furthermore it became evident that it would take too long for another trained person to get equipped with PPE to enter the laboratory for emergency care of injured RLWs. Therefore, all BSL-3 team members are trained in advanced life support. Emergency trainings consisted of first aid, emergency decontamination, and recovery of persons from the laboratory and are performed regularly and also include the rescue chain outside the laboratory, e.g., notification and instruction of the ambulance and the fire brigade.

## Discussion

The COVID-19 pandemic’s ongoing crisis has strained hospitals and health systems around the globe and upended culture, operations and management. While previous studies on preparedness and use of PPE did not receive enough attention at that time as evidenced by the insufficient readiness of PPE-trained personnel and availability of PPE, these types of studies have gained new relevance due to COVID-19 as so many HCWs and RLWs became infected and died (WHO, 2021).

## Lessons Learned From Previous Pandemics

Lessons learned from previous endemic diseases such as Ebola (MacIntyre et al., 2014a,b; CDC, 2015; Diarra et al., 2016; McLaws et al., 2016; Durski et al., 2017; Reidy et al., 2017; DuBose et al., 2018; Mumma et al., 2018, 2019; Lockhart et al., 2020), Crimean-Congo-Fever, Zika (Patel et al., 2021), SARS (Drosten et al., 2003; Rota et al., 2003; Berger et al., 2004) and MERS (Bermingham et al., 2012; Corman et al., 2012) were rediscovered and cited, and found their way into measures deployed to prevent SARS-CoV-2 transmission, underscoring the importance of proper use of PPE (Best and Williams, 2021; Lippi et al., 2020; Pillai et al., 2021; Verbeek et al., 2020) conclude that learning about the sourcing of PPE, supply management, leadership, learning and resilience was limited but has been increased by the SARS-CoV-2 pandemic regarding the importance of planning, collaboration and relationship building. They add that “*Resilience of PPE supply chains was reported to be dependent on multiple levels from individuals to organization level and also interdependent on (i) sustainability, (ii) the practice of PPE and (iii) long term environmental impact of PPE suggesting the need, long term, to move to a circular economy approach*” (Best and Williams, 2021).

Scientific knowledge about virus properties gained from SARS in 2003 and MERS in 2012 was used and re-evaluated for extraordinarily quickly research capacities for SARS-CoV-2 vaccine and drug development, and treatment initiatives (Tsang et al., 2003; Nicholls et al., 2006; de Wit et al., 2016).

## The Role of Personal Protective Equipment, Training, and Worker Protection Policies

Before the pandemic some hospitals equipped with established isolation units had teams that regularly trained on patient transport and care processes and how to best use PPE. However, no health care system expected the magnitude of personnel and PPE requirements and high consumption, nor the workload needed to contain SARS-CoV-2. As a consequence emergency training on PPE use had to be initiated rapidly according to the CDC guidelines (CDC, 2020, 2021; Auerbach et al., 2021). However, this required experienced trainers who were often not available. Research laboratories played an important role due to their ongoing experience working with high risk pathogens, and their well-trained RLWs familiar with the latest PPE and HCBL technologies in biosafety training. The support of experienced trainers was an additional resilience factor for the BSL-3 environment (see Table 2).

**TABLE 2:** Challenges due to supply bottlenecks, proposed solutions and questions.

Problem	Solution	This is what you need to pay attention to	Questions that arise.
Components with which you may have trained, were not available	Elaborate search for alternative products and order other components	Appropriate safety category	Are you familiar with this topics?
		Approval criteria	
		Certificates and norms	
		Fake certificates	How to identify fakes?
		Counterfeit products of low quality and insufficient protection	How to be sure of not ordering counterfeit products?
		Compatibility to existing components	How to ensure compatibility?
	Ask other organizations in the area if they can help out	Most of them are confronted with the same problem themselves	
Unprecedented price increase	Well organized stock piling	Storage capacity	Is there enough storage capacity?
	Observe the market	Time consuming	Do you need additional manpower to manage all personal protective equipment (PPE) issues?
How to continue safe patient care or lab work?	Off-label re-use of PPE components that are approved for single use	Develop a process for decontamination of contaminated PPE components	How to ensure sufficient decontamination?
			Which disinfectant should be used and what are the incubation times on different materials?
			Have you ever tested that?
			Are there already any data available?
			How often can you decontaminate until the material no longer has a protective effect?
		How and where to store components during decontamination?	Where to dry the PPE when space is limited?
		What are the legal consequences of off-label re-use?	Who decides about breaking rules, laws?
			How to minimize individual and general health and safety risks

PPE components are worn in multiple layers for personal protection in BSL-3 environments and require dedicated donning and doffing space and detailed processes. PPE typically consists of coveralls, available in different safety categories, liquid tight aprons, FFP-3 masks, goggles, and face shields or a protective hood with a PAPR, shoe covers, and gloves with different protection categories for different applications and working processes (Tables 3A, 3B and Figures 1A–D). Wearing PPE daily over many hours was extremely exhausting, and the strain varied depending on the components used. Heat stress, reduced dexterity and fine motor skills, sight and hearing restrictions, limited toilet use, and reduced liquid intake were evaluated in several studies (Garner et al., 2004; MacDonald et al., 2006; Kümin et al., 2011; O’Brien et al., 2011; Merens et al., 2012; Davey et al., 2021; Ruskin et al., 2021), including increases in cognitive load and enhanced medical errors (Zimmerman et al., 1991; Caretti, 1997, 1999; AlGhamri et al., 2013; Loibner et al., 2019).

**TABLE 3A:** Personal protective equipment (PPE) components used in the BSL-3 laboratory of the Medical University Graz for different PPE variants 1–4 (+yes, used; –no, not used).

	PPE variant 1	PPE variant 2	PPE variant 3	PPE variant 4
				
	Multi-layer with FFP3 mask	Multi-layer with PAPR	Liquid-tight with PAPR	Liquid-tight one-piece
Surgical scrub	+	+	+	+
Single-use coverall, Cat. III, type 4-B/5-B/6-B	+	+	–	–
Coverall category III, Type 3/4/5/6	–	–	+	+
Apron with long sleeves	+	+	–	–
Inner layer of gloves	+	+	+	+
Outer layer of chemically resistant gloves	+	+	+	Integrated with suit
Cut protection gloves	For autopsies	For autopsies	For autopsies*	For autopsies*
FFP3 or N100 mask	+	–	–	–
PAPR with A2BEKP filters	–	+	+	+
Eye goggles	+	–	–	–
Face shield	+	–	–	–
Croc shoes	+	+	–	+
Rubber boots	–	–	+	–
Double layer of shoe covers	+	+	–	–

*Optional, but currently not used.

**TABLE 3B:** The four different personal protective equipment (PPE) variants offer different levels of safety, usability, and personal perceptions.

	PPE variant 1	PPE variant 2	PPE variant 3	PPE variant 4
				
	Multi-layer with FFP3 mask	Multi-layer with PAPR	Liquid-tight with hood	Liquid-tight one-piece
Autopsy of deceased infected with a BSL-3 pathogen	*	**	***	***
Lab work with propagative BSL-3 pathogens	*	**	***	***
Strictly dedicated doffing process	Yes	Yes	No	No
Use of chemical shower possible	No	No	Yes	Yes
Heat stress	***	**	**	*
Extended working hours	*	**	***	**
Risk of slipping	**	**	No	*
Restricted fine motor skills	**	**	**	***
Restricted view	***	*	*	*
Foggy goggles	***	No	No	No
Use of adhesive tape to seal gloves, boots or shoe covers	Yes	Yes	Yes	No
PAPR battery exchange possible	Not used here	Yes	Yes	No

***Completely applies; **Applies well; *Applies to a limited extent.

## The Role of Supply Chains for Healthcare and Research Laboratory Operations

The role of supply chains, equipment procurement, and the dependency on manufacturers and suppliers of PPE components, disinfectants, and laboratory material was grossly underestimated by healthcare systems and research organizations (Burnett et al., 2022). HCWs and RLWs faced the additional situation of global delivery stops and delays of several months for PPE components in the early stage of the pandemic (Antonini et al., 2021; Best and Williams, 2021; Kothakonda et al., 2021; Ogbuoji et al., 2021; Pillai et al., 2021; Plana et al., 2021; Weaver et al., 2021; Loibner et al., 2021b). The three major problems of PPE unavailability, unprecedented rise in prices and maintenance of safety are described in Table 4. The proposed solutions raise many questions that need to be addressed locally and internationally for sustained preparedness for future pandemics.

**TABLE 4:** Key lessons learned from BSL-3 environment, recommendations and policy implications.

Problems encountered	Recommendations laboratory level	Recommendations organizational unit level	Recommendations responsible policy level
Disrupted personal protective equipment (PPE) supply chain	See Table 4	Support through more man power, forward-looking stockpiling at a higher management and supply level	Establish a national focal point for capturing PPE needs in health care and research institutions, and rapid evaluation of the needs
		Rapid demand forwarding to national focal point	Securing a national supply for patient care and laboratories
Correct use of PPE	Laboratory specific training	Awareness and support for training through adjustment of duty times for training sessions	Awareness and support to implement biosafety and PPE into educational programs for nurses, physicians, technicians, lab workers, natural scientists
			Adaptation of national legislations for PPE according to the biosafety level of the respective facility
High physical strain due to PPE	Feedback to the manufacturers regarding physical, ergonomic, biosafety, and scientific requirements	Providing funding for R&D programs for developing better equipment	Adaptation of the legal bases and resilience limits especially for high-security laboratory work within the Employee Protection Act (with regard to ergonomics, temperature, physical and mental strain) also with regard to salary grades
	Individual fit i.e., length and width of the suit, slip-resistant soles	Call to manufacturers for more innovative and appropriate products	
	Better temperature perception and sweating due to more appropriate material and textiles, and suit ventilation (including PAPR easy battery change)		
High workload and personal resilience	Reasonable selection and prioritization of work packages and adaptation to the available manpower	Merging of trained staff from other less burdened units; this requires harmonized and interoperable procedures	Awareness and support to implement biosafety and PPE into educational programs for nurses, physicians, technicians, lab workers, natural scientists to increase the number of trained personnel
	Take into account staff absences due to infections, quarantine, and breaks and recovery times	Train sufficient personnel and keep them on standby	
	Train sufficient number of personnel and keep them on standby through regular outreach	Recognition and financial and time compensation for the high physical stress and risk of infection when working with PPE	
	Reduced distress due to several years of experience, routine, biosafety trainings (including emergency and spill trainings)	Joint trainings (biosafety, emergency, spill, etc.) with different departments and organizations	
Lack of trained personnel	Laboratory specific training	Retain trained personnel and capacities even in non-crisis times through continuous research projects	Continuous financial, economic, and educational support for research projects in HCBLs
			Secure and hard funding is needed to sustain HCBLs and their personnel

The pandemic also uncovered parochial national interests such as delivery embargos, customs and border blockades that were experienced not only for PPE, but also for essential research reagents (e.g., kits for virus RNA isolation and qPCR), disinfectants, and reference materials (e.g., cell lines, viruses). This situation hindered clinical management, research and diagnostic work and also caused considerable stress for employees and endangered HCW and RLW. Detailed strategies for rational use and sharing of PPE supplies published by Mahmood et al. (2020) in May, at the time of the greatest supply bottlenecks, recommended the necessity of (off-label) re-use of PPE in spite of the inherent contamination risks to HCW (Mahmood et al., 2020). We know that the reuse of PPE endangers HCW and recent studies have shown significant contamination with reuse of PPE (Doos et al., 2022).

## Occupational Working Conditions With Personal Protective Equipment

In general, four continuous working hours wearing PPE are considered to be well tolerated under ambient temperatures of 20°C and also reported to be strictly controlled in some institutions (Lin et al., 2020). Longer working shifts, however, became routine in most hospitals and directly increased the risks of respiratory and circulatory problems (Carter and Notter, 2020; Davey et al., 2021) as well as anxiety and human errors (Alarfaj et al., 2021; Doos et al., 2022). A survey among 224 HCWs wearing PPE revealed that 27% worked for 0–4 h, 34% for 4–8 h, 33% for 8–11 h, and 6% worked more than 12 h, which far exceeds all recommendations and underlines the enormous challenges to healthcare operations posed by the COVID-19 pandemic (Davey et al., 2021). Extended working periods and increased workloads brought HCWs, RLWs, and their managers into conflicts with European Working Hour laws and statutory rest periods (European Union, 2021). HCWs had to go through the risky process of PPE doffing, including changing their PPE several times in order to prevent viral transmission between infected patients. During these doffing changes short breaks were taken for body evacuation needs at a minimum due to time pressure working in overcrowded wards. One of the few publications about RLWs reports that 67.7% of 7,911 qualified biosafety laboratory staff in China experienced job burnout, with a particular higher risk for post-graduate women, aged 45–50, with 11–20 years of experience (Lu et al., 2021). Medical scientists in Nigeria reported in an online survey that their awareness for laboratory biosafety was at 60.3% which significantly corresponded to their education level, but only 45.1% attested to the availability of adequate PPE including training, adequate rest and access to biosafety cabinets (Lekan-Agunbiade and Agunbiade, 2021). Emerging problems can lead to further risks of damage to both physical and mental health as stated by Di Prinzio et al. (2021). They suggest involving assistance persons, providing specific training, and a proper rest and turnover of personnel to improve coping skills and resilience of HCWs (Di Prinzio et al., 2021). These recommendations are consistent with our findings (see Table 2).

## Role of Training for Personal Protective Equipment Competencies

Good training for regular work but also preparation for unpredictable situations are key for PPE users, especially for the doffing process. This has been well known for the past 2 decades from Ebola outbreaks (CDC, 2015; McLaws et al., 2016; Casanova et al., 2018; Mumma et al., 2018, 2019; Wong et al., 2019) and was confirmed during the SARS-CoV-2 pandemic (McCarthy et al., 2020; Wong et al., 2020; Yuan et al., 2020a,b). However, in spite of this long standing knowledge few HCWs had been trained on detailed PPE competencies. Candiotti et al. (2005), found that only 37% of HCWs had any form of training, and many of them did not repeat training after initial sessions.

HCWs and RLWs had to quickly react to the changing working conditions, i.e., the lack of PPE components and proper training. The training philosophy “*train not until you get it right but until you never get it wrong*” is essential but could not be implemented due to time pressures and significant PPE shortage at the beginning of the pandemic. Picard et al. (2021) published an analysis of the role of trained observers, so-called “dofficers,” to decrease the high error rates during PPE doffing processes via a “dofficer” that included a 21-point audit. The insights by dofficers resulted in identification of areas that needed to be improved and further investigated for their causality (Devin et al., 2021; Picard et al., 2021) demonstrated that in a simulation study involving the examination of five consecutive patients, nearly all HCWs asked to don and doff PPE per CDC requirements repeatedly contaminated themselves (Devin et al., 2021). Hick and Thorne summarized the urgency surrounding PPE issues in 2006(!) addressing the types, use, selection, and decontamination and stated: “*We can only hope that we are not forced to learn too many more harsh lessons about PPE use in the future. In the meantime, however, we should strive to prepare our communities by selecting appropriate protective technologies in relation to perceived threats and practicing our responses so that our personnel are comfortable using their PPE and understand the consequences of not doing so”* (Hick and Craig, 2006). A survey among 653 RLWs in Blood Transfusion departments published by Liu et al. (2021) revealed much vague awareness and as many as 4.7% did not receive any safety and biosecurity training. The major deficiencies were suboptimal safety practices and laboratory conditions (Liu et al., 2021).

The scientific capabilities of well-trained personnel working in HCBLs are a tremendous resource not only because of the research they conduct, but also because they provide critical guidance and leadership on how to safely manage highly infectious pathogens. Nyaruaba et al. (2021) summarized the current SARS-CoV-2 laboratory biosafety practices and current molecular diagnostic tools and their impact to combat future outbreaks. They called on the WHO and CDC to continuously update and revise biosafety protocols and techniques (Nyaruaba et al., 2021). The importance of comprehensive decontamination measures was underlined by Brandner et al. (2022), who reported that 41 of 198 (20%) samples taken from PPE components after full autopsies tested positive for SARS-CoV-2 RNA, with 64% of gloves, 50% of aprons, and 36% of tops of shoes, and 27% of these samples were still infectious (Brandner et al., 2022).

We recommend that all RLWs undergo testing of their PPE under the expected working conditions in a non-infectious environment to test the proper function of PPE and to determine whether they can tolerate all PPE-related restrictions, before working with pathogens in a BSL-3 laboratory. Raising awareness for the appropriate use of PPE, especially the hazardous doffing process, is key as this bears a high risk for contamination and infection (Carter and Notter, 2020; Huang et al., 2020; Lin et al., 2020; Lockhart et al., 2020; McCarthy et al., 2020; Verbeek et al., 2020; Yuan et al., 2020a; Ruskin et al., 2021; Tang et al., 2021). Using checklists and regular training for donning and doffing processes should be mandatory in order to reduce risks and to avoid errors (Lockhart et al., 2020; McCarthy et al., 2020; Ruskin et al., 2021). Heat stress, high workload and dexterity challenges can be well simulated in trainings (Wong et al., 2021). Factors that cannot easily be simulated include the mental stress, such as addressing the deep anxiety of working with highly infectious pathogens, being exposed to the caustic acid in the chemical shower, injury of team members or other emergency situations like fire.

## Risk Management in High Containment Biological Laboratories

Risk is defined as exposure to potentially injurious events that may threaten or damage the individual or an organization (Friedberg and Barach, 2018). Risk analysis applied to COVID-19 and PPE provides a valuable framework to help understand the potential dangers of communicable diseases and weigh the options to help HCW and RLW stay safe while navigating their choices. It includes the following three key steps: risk assessment, risk management, and risk communication. These scientific tools can help assess threats to human health, provide input into how to manage these risks, and communicate more effectively with the general public about how best to respond to these threats. With COVID-19 it is clear that how we assess and manage risk is important to guiding policies that reduce disease transmission (Romani et al., 2021).

The Graz BSL-3 laboratory implemented the detailed documentation of all incidents and near misses including corrective and prevention measures as well as suggestions for improvements independent of incidents as part of the laboratory risk management procedures (see also ISO 35001:2019). This documentation of even minor process failures that are typically neglected generates an opportunity to raise awareness of incidents that may happen and that one would never expect (i.e., “all that can go wrong will go wrong; it is just a matter of time”). The detailed documentation and assessment of incidents demonstrated the importance of designing safety management systems and processes for redundancy of procedures and safety systems allowing failure at one level to still be compensated by other levels (Sanchez and Barach, 2012). As a consequence none of the incidents reported led to infection or major injury of a BSL-3 team member or posed any risk to the environment. This safety culture based on transparent failure and robust risk management practices led to continuously improved procedures and increased trust within the team whose safety critically relies on each other (Bognár et al., 2008).

Another key tool to addressing the risks posed to HCWs and RLWs in dealing with COVID-19 patients is the failure mode and effect analysis (FMEA), a systematic approach for identifying all possible failures in a design, in the manufacturing or assembly process, or in the product or service (Loibner et al., 2021b). FMEA was first developed by the US military in the 1940s and was adopted by the National Aeronautics and Space Administration (NASA) in connection with attenuating risks of manned space missions (mid-1960s). Widely practiced throughout the automotive, software, food services and many other industries, only in recent years has FMEA been successfully applied in healthcare as a proactive tool to improve patient safety and efficiency in hospitals (Cheng et al., 2012; Toccafondi et al., 2021). FMEA is regularly used for risk assessment of biopharmaceutical manufacturing processes, analytical procedures for screening drugs and more recently in clinical trials (van Tilburg et al., 2006; Stojković et al., 2017).

## Policy Questions – WHO, CDC, EU – Guidance

The HCBL minimum design requirements are defined by the WHO Guideline “Infection prevention and control of epidemic- and pandemic-prone acute respiratory infections in health care” (WHO, 2014), the Laboratory Biosafety Manual Fourth Edition published on 21.12.2020 (WHO, 2020b), and the “Directive 2000/54/EC of the European Parliament and of the Council of 18 September 2000 on the protection of workers from risks related to exposure to biological agents at work” with its consolidated version of 24.06.2020 (European Union, 2020). National legislation e.g., in Austria, does not further specify the PPE components to be used in HCBL/BSL-3 environments, stating only that PPE has to be “adequate” or “appropriate,” which is vague and leaves most of the concerns detailed above unaddressed.

New guidelines were created and implemented during the pandemic due to PPE shortages. Antonini et al. (2021) reported on the rapid manufacturing of face shields via a design and product development framework with varied stakeholders including clinicians, healthcare facility managers, infection control specialists at CDC, local suppliers, manufacturers, and the CDC guidance allowing for extended use, re-processing and re-use of PPE (CDC, 2008; Antonini et al., 2021). However, even when in compliance with CDC and FDA guidelines designers must ensure the compatibility of the PPE products with decontamination needs and anticipated sterilization and empirical testing (Antonini et al., 2021; Plana et al., 2021) reported that they tested over one hundred N95 masks (under normal circumstances they have 2–5 models in their local inventory) after the Emergency Use Authorization (EUA) from the FDA (FDA, 2020; Plana et al., 2021), and found that many of those were not correctly labeled (CE, NIOSH), and did not perform to specific safety and engineered standards. Some were obviously dangerous, and many were likely counterfeits. Masks claiming multiple non-identical regulatory approvals were fraudulent, e.g., Chinese KN95 masks were labeled with CE and NIOSH logos or PM 2.5 masks were fraudulently labeled with N95 or KN95 on their packaging (Plana et al., 2021).

## Acknowledgments of Conceptual or Methodological Limitations

This manuscript is centered on a case study describing the authors’ experience of RLWs in the BLS-3 laboratory at the Medical University Graz during the COVID-19 pandemic. Therefore, the experience obtained and lessons learned may not be generalizable. However, the broad spectrum of different types of research work performed in this laboratory might be of relevance in a wider context. This is substantiated by the extensive review of other studies and experience obtained by HCWs, which demonstrated agreement with several of our observations generated in the BSL-3 laboratory. Since BSL-3 laboratories may also have a broad spectrum of different designs and have implemented different procedures these specific aspects should be considered in drawing general conclusions.

## Conclusion and Recommendations

The COVID-19 pandemic resulted in unprecedented organizational, physical and emotional challenges for HCWs and RLWs and health system operations. Our experience demonstrated significant limitations of the current PPE solutions and highlighted the urgency for innovation and improvement of PPE design and protocols. Future similar or even worse pandemics are likely and we must harvest the maximum insights from the current situation. Although several studies have investigated the challenges and pitfalls of protecting HCWs this is one of the few reporting on the lived, real world experience of HCBL and RLWs during COVID-19.

The main take home messages can be summarized as follows:

i) Pandemics create a tremendous increase in workload which in combination with shortage in PPE components and research reagents requires robust, flexible, and reliable processes that can be adapted to new operational demands.
ii) Redundancy in safety solutions, flexibility in the composition and use of PPE components are essential.
iii) Problems observed with current PPE components indicate significant opportunities for redesign and should stimulate industry to invest in developing and testing innovative products.
iv) A cornerstone in achieving resilience and proper protection of HCWs and RLWs is regular training and competency improvement.
v) A dedicated risk and failure management system and culture have to be implemented in all healthcare and research facilities that continuously feeds into adaptive practices to be directly included in training programs.
vi) A chemical shower, which is a requirement for BSL-4 laboratories (and not needed for BSL-3 laboratories), proved to be very useful not only for decontamination of corpses after autopsy but also for decontamination of reused PPE by RLWs. This on the one hand reduced the risk of contamination of RLWs during PPE doffing, and on the other hand allowed re-use of PPE components safely, which was essential to stay operational during the first phases of the pandemic.
vii) Simple, low-cost environmental design interventions can provide structure to support and improve HCW/RLW safety in HCBS (DuBose et al., 2018).

## Author Contributions

ML, PB, and KZ contributed to conception and design of the manuscript. ML wrote the first draft of the manuscript. PB and KZ wrote sections of the manuscript. ML, SW, CL, VS, JR, EF-H, MH, EK, PW, MZ, SG, GG, and PR provided experience from research work or autopsies performed in the BSL-3 facility. All authors contributed to manuscript revision and approved the submitted version.

## Conflict of Interest

The authors declare that the research was conducted in the absence of any commercial or financial relationships that could be construed as a potential conflict of interest.

## Publisher’s Note

All claims expressed in this article are solely those of the authors and do not necessarily represent those of their affiliated organizations, or those of the publisher, the editors and the reviewers. Any product that may be evaluated in this article, or claim that may be made by its manufacturer, is not guaranteed or endorsed by the publisher.

## Footnotes

1
www.erinha.eu
